# The Swallowable Intragastric Balloon Combined with Lifestyle Coaching: Short-Term Results of a Safe and Effective Weight Loss Treatment for People Living with Overweight and Obesity

**DOI:** 10.1007/s11695-023-06573-8

**Published:** 2023-04-03

**Authors:** Marijn T.F. Jense, Inge H. Palm-Meinders, Boy Sanders, Evert-Jan G. Boerma, Jan Willem M. Greve

**Affiliations:** 1 Bariatric Surgery, Zuyderland Medical Center, Henri Dunantstraat 5, 6419 Heerlen, PC Netherlands; 2 Dutch Obesity Clinic South, John F. Kennedylaan 301, 6419 Heerlen, XZ Netherlands; 3 Maastricht University Medical Center, Maastricht, The Netherlands

**Keywords:** Intragastric balloon, Obesity treatment, Obesity, Overweight, Weight loss, Lifestyle coaching

## Abstract

**Background:**

Some patients with overweight or obesity are not eligible for surgery according to international guidelines or do not wish a surgical intervention. For these patients, different treatment options are being explored. In this study, we examined the effectiveness of the swallowable intragastric balloon (IB) combined with lifestyle coaching, in patients living with overweight and obesity.

**Method:**

A retrospective data study was conducted on patients with a swallowable IB placement between December 2018 and July 2021, combined with a 12-month coaching program. Before balloon placement, patients underwent multidisciplinary screening. The IB was swallowed and filled with fluid once in the stomach and naturally excreted around 16 weeks.

**Results:**

A total of 336 patients, 71.7% female, were included with a mean age of 45.7 (±11.7) years. Mean baseline weight and BMI were 107.54 (±19.16) kg and 36.1 (±5.02) kg/m^2^. After 1 year, the mean total weight loss was 11.0% (±8.4). The mean placement duration was 13.1 (±2.82) min, and in 43.7%, a stylet was used to facilitate placement. The most common symptoms were nausea (80.4%) and gastric pain (80.3%). In the majority of patients, complaints were resolved within a week. The early deflation of the balloon occurred in 8 patients (2.4%) of which one showed symptoms suggesting a gastric outlet obstruction.

**Conclusion:**

Given the low rate of long-term complaints while providing a positive effect on weight loss, we conclude that the swallowable intragastric balloon, combined with lifestyle coaching, is a safe and effective treatment option for patients living with overweight and obesity.

**Graphical Abstract:**

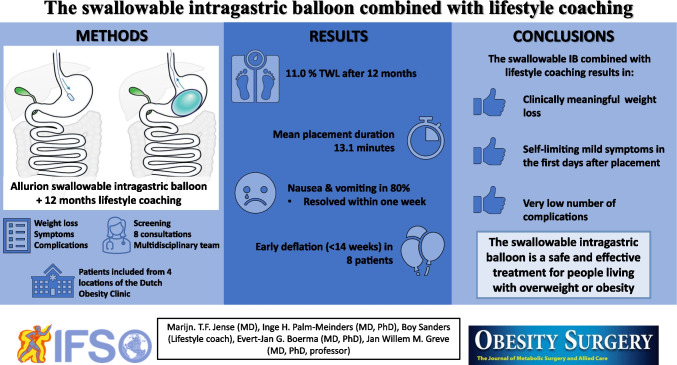

## Introduction

For people living with obesity, bariatric surgery is a possible solution. In The Netherlands, bariatric surgery is currently only reimbursed for patients who comply with international guidelines stating that a patient needs to have a BMI of at least 35 kg/m^2^ with comorbidities or a BMI of 40 kg/m^2^ [1, 2]. However, some patients are not eligible for surgery according to these international guidelines or do not wish a surgical intervention.

Other treatment options, such as anti-obesity medication (AOM) or the intragastric balloon (IB), are being explored. Since the beginning of 2022, certain types of AOM are available as an insured treatment option in combination with intensive lifestyle coaching in The Netherlands [3, 4]. Although multiple pharmacological agents have been proven to be effective for weight loss, they do come with side effects. In addition to the side effects, interactions with regularly used medication are common and worrisome [5–7].

The use of IBs has already been reported [8]. However, most IBs have to be placed endoscopically [8, 9]. Although endoscopy is less invasive than surgery, it remains to be invasive. The difference with the Allurion IB is that this balloon has to be swallowed by the patient. Once the capsule with the balloon is in the stomach, the balloon is filled with fluid. After 16 weeks, a filament attached to the valve of the balloon will be dissolved. Once the filament is dissolved the valve will open and the balloon will empty itself in the stomach. The fluid and the emptied balloon will be excreted via the intestinal tract. This way, no endoscopy is necessary, and therefore the treatment is less invasive, time-consuming, and costly.

In the Dutch Obesity Clinic (DOC), one of the treatment options is 12 months of lifestyle coaching combined with an Allurion IB. The patients have to pay for the treatment themselves. Although multiple studies have reported on the use of the Allurion IB, not many studies have combined the use of the IB with lifestyle coaching for a longer period [10, 11]. Via the current retrospective data study, we aim to evaluate the safety and effectiveness of the Allurion IB in combination with lifestyle coaching.

## Method

### Study Design

All patient data used in this study were obtained from patients who have received an IB at the DOC and have given their consent to use patient data for scientific research. In addition to the IB, patients received lifestyle coaching to be able to maintain their weight loss. For this retrospective data study, local approval was given by the ethics committee in accordance with the ethical standards as laid down in the 2013 Declaration of Helsinki. All data were collected and blinded in SPSS by a health professional of the DOC who had a treatment relationship with the patients. The researcher who worked with the data was not able to trace information back to the patients.

### Participant and Eligibility Criteria

The study population consisted of patients who received a balloon between December 2018 and July 2021. All adult patients who were interested in obesity treatment and had a BMI above 27 kg/m^2^ were screened by the medical doctors of the DOC. Pregnancy, previous operations in the upper gastro-intestinal region, and trouble with swallowing were the main exclusion criteria. Additional exclusion criteria are listed in Appendix 2. If the patient was considered eligible, treatment with an IB was initiated. Included in the treatment with the Allurion balloon at the DOC was a 12-month lifestyle program.

### Placement

The IB is swallowed by the patient and filled with 550 mL fluid, after confirmation of the intragastric position via X-ray. After 16 weeks, the balloon deflates spontaneously and leaves the body through the intestinal tract. The duration of every placement and the use of a stylet (guidewire) were recorded at every placement appointment.

### Comorbidities

Hypertension, type 2 diabetes, dyslipidemia, and obstructive sleep apnea syndrome (OSAS) were assessed during screening. For the definitions of comorbidities, the criteria described by the ASMBS were used [12].

### After Placement

One day and 1 week after balloon placement, all patients were contacted via telephone. After 1 week, they were questioned about nausea or vomiting, pain, or cramps during the first days after placement of the balloon and whether they have had to take the prescribed spasmolytic drug to ameliorate cramps. Furthermore, all patients were asked if they complied with the use of a proton pump inhibitor (PPI), drank enough fluids, followed the prescribed diet, experienced satiety between meals, and within how many days they returned to work.

The lifestyle coaching program consisted of 8 consultations with a dietitian, psychologist, or medical doctor during the 12 months after placement. The focus and subject of these consultations are planned according to the needs and wishes of each individual patient. Possible subjects include eating habits, activity, sleep habits, coping with stress, emotional regulation, and other factors that possibly have an impact on one’s weight. One year after balloon placement, all patients were contacted for a final consultation via a telephone call. If patients did not respond after multiple reminders, data was recorded as missing.

### Statistical Analysis

Statistical analysis was performed using version 26 of SPSS software. Firstly, all results were tested for normality using the Kolmogorov-Smirnov test. Categorical variables were expressed as *n* (%), continuous normally distributed variables by their mean and standard deviation, and not normally distributed data by their median and interquartile range.

## Results

Between December 2018 and July 2021, 336 patients were included of whom 71.4% were female. At screening, the mean age was 45.7 years (SD ±11.7), and the mean BMI was 36.1 kg/m^2^ (SD ±5.0) (Table 1).

### Loss to Follow-Up

At all 3 time points, different percentages of patients were lost to follow-up, ranging from 10.1 to 57.1%. In all tables and figures, the amount of missing data per measurement and time point is described.

### Placement

The majority (81%) of the IBs were placed at one location of the DOC. The mean duration of the placement was 13.05 (±2.81) with a minimum of 8 and a maximum of 27 min. In 143 patients (43.4%), a guidewire (stylet) was used to help with the placement (Table 2).

### Complaints and Symptoms

After balloon placement, the majority of patients (80.4%) suffered from nausea or vomiting and pain was present in 80.3% of the patients. All these symptoms responded to medical treatment and were resolved within 1 week. In 5 patients (1.48%), the balloon had to be extracted endoscopically because of intolerance causing severe nausea and vomiting. In one of these five patients, a clear obstruction of the pylorus causing food blockage in the stomach occurred.

Most patients (85.6%) managed to follow the diet according to protocol. Fluid intake of > 1.5 L per day in the first week was successful in 77.3% of the patients. In 90.3% of patients, the balloon caused satiety between meals. After placement, the mean time to return to work was 3.94 (±2.31) days (Table 3).

### Early Deflation

In 8 (2.38%) patients, the balloon deflated before 14 weeks. In one of these 8 patients, the balloon had to be removed endoscopically after an early partial deflation, causing symptoms suggesting a gastric outlet obstruction. All eight patients were offered a free sequential balloon, and 5 of these patients chose to receive this sequential balloon.

### Weight Loss

**Fig. 1:**
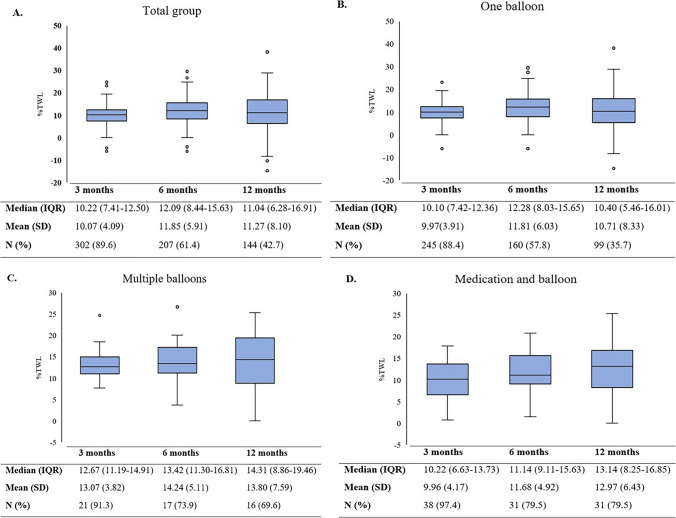
Percentage of total weight loss over time for all different groups. **A** Total study group. **B** Patients who received one balloon. **C** Patients who received multiple balloons. **D** Patients who received additional medication with or after the balloon. IQR interquartile range, SD standard deviation

When studying the results of people who received one balloon without any additional treatment, such as sequential balloon or weight loss medication, the %TWL at 3 months was 9.94 (±3.98). At 6 months, a %TWL of 11.83 (±5.99) was demonstrated, and at 12 months, the %TWL was 10.97 (±8.39). Some patients decided to have sequential balloons or combine the treatment with the use of medication. The TWL results of these patients were analyzed separately (Fig. 1).

After 3 months, the mean percentage of total weight loss (%TWL) of the total study group was 10.07 (SD ±4.09). After deflation of the balloon at 4 months, the mean %TWL continued up to 11.85 (± 5.91) at 6 months and then slightly decreased to 11.27 (±8.10) at 12 months.

### Combined Treatment

As described in the weight loss section, multiple patients had additional treatment with either a sequential IB or AOM. A sequential balloon was chosen by 23 patients (6.85%), and AOM was chosen by 39 patients (11.6%). People who received a sequential balloon had a %TWL at 3 months of 13.07 (3.82), 14.24 (±5.11) at 6 months, and 13.80 (±7.59) at 12 months. The time between the balloon and a sequential balloon was 28.7 weeks on average, with a minimum of 4 and a maximum of 119 weeks. The %TWL between the first balloon and the sequential balloon was −3.0 (±5.6) on average.

Liraglutide and naltrexone/bupropion were the AOM of choice with the majority of patients (89.7%) choosing liraglutide. When combining the balloon with medication, the results were a %TWL of 10.03 (±3.66) at 3 months, 11.51 (±4.94) at 6 months, and 12.59 (±6.48) at 12 months.

The time between the balloon and the start of additional AOM was 29.1 weeks after balloon deflation on average, with a minimum of −9 weeks and a maximum of 128 weeks. The subzero numbers mean that a patient started with the medication while the balloon was still in place.

The timing of starting additional treatment was at the discretion of the patient, for instance, because of increased appetite.

## Discussion

The swallowable intragastric balloon combined with lifestyle coaching can be effectively used as weight loss treatment for people living with overweight or obesity. Our study presented clinically meaningful weight loss results at 12 months follow-up, which are comparable to the results of previous studies on smaller groups [13, 14]. In studies which describe the weight loss results after the 16 weeks of balloon treatment, we see comparable results as well of TWL between 10 and 14% [11, 13–15].

The dwell time of around 16 weeks of the Allurion balloon has been named as one of the disadvantages of this type of IB. However, balloons with a dwell time of 12 months show similar weight loss results to our study [16]. Furthermore, 80% of the weight loss happens in the first 3 months, as can be seen in this study’s results and the study by Gaur et al. [17].

The shorter dwell time also has an advantage: the balloon has less effect on the stomach wall, resulting in less risk for ulceration or pressure trauma on the stomach wall. These traumas can be seen with other balloons with a longer dwell time [18].

Although rare, balloon-related complications can occur. In our study population, 5 patients had to have the balloon removed via endoscopy due to intolerance symptoms, such as severe vomiting or eating difficulties. In one of these patients, a clear obstruction of the gastric outlet occurred. This form of obstruction has also been described by Ciprian et al. [19] as a rare but possibly dangerous complication. Another case report described an obstruction of the small bowel after the deflation of the balloon [20]. Fortunately, these complications are rare, especially when contraindications are followed strictly.

Additional lifestyle coaching to the balloon placement might be essential for successful weight loss results. Since the balloon will deflate after 16 weeks, the physical effect of the balloon will no longer be there. However, by combining the balloon with a 12-month lifestyle coaching program, the results achieved with the balloon can be maintained or even improve, as can be seen in this study’s results. Unfortunately, most studies on IBs do not include lifestyle coaching or do not compare the treatment with or without a lifestyle coaching [21]. One study does describe the difference in weight loss results of lifestyle coaching compared with a lifestyle coaching combined with IB placement, with a more positive effect in the group with combined treatment [22].

Even with lifestyle coaching, weight recurrence can occur after the balloon is deflated. Our study illustrates a possible solution. The use of AOM can help in maintaining weight loss results and can sometimes even result in additional weight loss. The combination of AOM with the IB has not been described sufficiently yet but has been suggested by several studies as a potential subject for research [23].

The weight loss effect of one balloon can be seen as a kick-start for patients to lose more weight without additional treatment. From patient experience, we know that these first kilos of weight loss can function as motivation to lose more weight. This is also described in a study focused on a group of adolescents with obesity who were treated with the IB and lifestyle coaching [24]. However, it is unclear how long this incentive lasts.

The use of sequential balloons did not show further weight loss on average. Besides, from our patient’s experience, sequential balloons could even cause more symptoms than the first balloon. The placement itself was not experienced as more difficult. Current literature does describe the use of sequential balloons in line with our results [25, 26]. On the other hand, Genco et al. describe a significant additional effect of sequential balloons. These results are however from a different type of balloon which remains in the stomach for 6 months [27].

The mean %TWL stabilized over 12 months. The stabilization of weight is of course a positive effect. However, if the patient who requested an additional treatment wants to lose additional weight or has weight recurrence after balloon deflation, an additional form of treatment should be evaluated thoroughly taking into account the treatment wish of the patient and the timing after the first balloon deflation.

A limitation of this study is that the patient group included is a group of people with the resources to pay for this form of treatment. This creates a bias in the representation of our patient group. Furthermore, when people have to pay for their treatment, they tend to be more committed to the entire treatment which can lead to a falsely high follow-up and compliance rate [28]. However, in the current study, a loss to follow-up rate was seen of 10.1% to 57.1%, which can be seen as a limitation of this study.

## Conclusion

Given the low rate of long-term complaints while providing a positive effect on weight loss, we conclude that the swallowable intragastric balloon, combined with lifestyle coaching, is a safe and effective treatment option for patients living with overweight and obesity. In addition, the intragastric balloon can be combined with weight loss medication for additional weight loss if needed.

## Appendix 1

**Table 1:** Baseline characteristics as reported during screening

		Count (*N* = 336)	Minimum–maximum
Sex	Female	241 (71.7 %)	
	Male	95 (28.3 %)	
Mean age (SD)		45.7 (± 11.7)	18–75
Mean weight in kg (SD)		107.54 (± 19.16)	64.90–180.00
Mean BMI in kg/m^2^ (SD)		36.10 (± 5.02)	27.14–57.77
Hypertension		64 (19.5%)	
Diabetes mellitus		16 (4.8%)	
Dyslipidemia		33 (9.8%)	
Obstructive sleep apnea syndrome		45 (13.4%)	

*SD* standard deviation, *kg* kilograms, *BMI* body mass index

**Table 2:** Balloon placement

		Count (*N* =336)	Minimum–maximum
DOC location	Gouda	64 (19%)	
	Heerlen	272 (81%)	
Mean duration of treatment in minutes (SD)		13.05 (± 2.81)	8–27
Use of stylet for placement		145 (43.4%)	

*DOC* Dutch Obesity Clinic, *SD* standard deviation

**Table 3:** Symptoms first week after balloon placement

		Count	*N* %	Missing	Min.–max.
Nausea or vomiting		251/312	80.4%	24	
Pain		245/305	80.3%	31	
Buscopan needed		239/294	81.3%	42	
Daily use of PPI		293/300	97.7%	36	
Mean days return to work (SD)		3.94 (2.32)			0–21
Diet conform protocol		255/298	85.6%	38	
Eating pattern	Irregular	25	12.8%	140	
	Regular	171	87.2%		
Fluid intake >1.5 L		231/299	77.3%	37	
Satisfied between meals		260/288	90.3%	48	
Extraction balloon due to complaints		5/336	1.48%	-	

*PPI* proton pump inhibitor, *SD* standard deviation, *L* liter

## Appendix 2

**In- and exclusion criteria Allurion intragastric balloon treatment**

*Inclusion*

- ≥ 18 years old

- BMI of ≥ 27 kg/m^2^

*Exclusion*

Difficulty swallowing (dysphagia):

• Any abnormal swallowing mechanism from an esophageal motility disorder such as achalasia, scleroderma, or diffuse esophageal spasm

• History of any structural esophageal abnormality such as a web, stricture, diverticulum, or para esophageal hernia

Conditions that predispose to bowel obstruction:

• History of perforated appendicitis or any other perforated abdominal viscus

• Crohn’s Disease

• Severe GI motility disorder such as severe gastroparesis

• Any history of actual, or suspected, bowel obstructions or small bowel surgery

• Any history of intraperitoneal adhesions

Conditions that predispose to gastric perforation:

• History of any previous bariatric, gastric or esophageal surgery

• History of previous laparoscopic band ligation

• History of anti-reflux surgery

GI bleeding or conditions that predispose to GI bleeding:

• Recent history of inflammatory conditions such as esophagitis, gastritis, gastric ulceration, or duodenal ulceration

• History of vascular lesions such as esophageal varices, gastric or duodenal varices, or intestinal telangiectasias

• Benign or malignant gastrointestinal tumors

• Inability to discontinue the use of non-steroidal antiinflammatory drugs (NSAIDs) or other gastric irritants during the device period

• Patients receiving anticoagulants

• Severe coagulopathy

• Hepatic insufficiency or cirrhosis

• Inability or unwillingness to take prescribed proton pump inhibitor medications in preparation for and/or during device residence

Other conditions:

• Serious or uncontrolled psychiatric illness

• Diagnosed bulimia, binge eating, compulsive overeating, or similar eating-related psychological disorders

• Alcoholism or drug addiction

• Pancreatitis

• Symptomatic congestive heart failure, cardiac arrhythmia, or unstable coronary artery disease

• Pre-existing significant respiratory diseases such as chronic obstructive pulmonary disease (COPD), severe sleep apnea, or cystic fibrosis

• Cancer

• Known or suspected allergies to polyurethane

• Inability or unwillingness to take prescribed antiemetic medications in preparation for and/or during device residence

• Women who are pregnant or nursing

• An existing gastric balloon that is currently in the stomach
